# Early blood pressure outcomes after SPYRAL™ renal denervation: an exploratory small cohort analysis

**DOI:** 10.3389/fcvm.2026.1934448

**Published:** 2026-10-01

**Authors:** Sasho Stanisavljevikj, Ali Anwar, Ahmed Abu Rumman, Hristijan Alagjozovski, Vladyslav Kavalerchyk, Ivica Bojovski, Davor Vukadinović, Joachim Weil, Alexander Staudt

**Affiliations:** 1 Friedrich-Alexander-Universität Erlangen-Nürnberg, Erlangen, Germany; 2 Die MSH Medical School Hamburg–University of Applied Sciences and Medical University, Hamburg, Germany; 3 Clinic for Cardiology and Angiology, Helios Clinics Schwerin, Schwerin, Germany; 4 University Clinic of Cardiology, Skopje, North Macedonia; 5 Department of Internal Medicine III–Cardiology, Angiology, and Internal Intensive Care, Saarland University Medical Center, Homburg, Saarland, Germany; 6 Med. Klinik II-Kardiologie und Angiologie, Sana Kliniken, Lübeck, Germany

**Keywords:** DBP (diastolic BP), RDN, resistan hypertension, SBP, spyral

## Abstract

**Background:**

Catheter-based renal denervation (RDN) is an interventional therapy for patients with resistant or uncontrolled arterial hypertension after the exclusion of secondary causes. This study aimed to evaluate the effects of the SPYRAL™ catheter system on systolic (SBP) and diastolic blood pressure (DBP) in a real-world clinical setting. Additionally, we explored whether the number of ablation points was associated with the magnitude of blood pressure (BP) reduction.

**Methods:**

We conducted a retrospective, single-center study including patients aged ≥18 years with predominantly resistant hypertension with preserved renal function (eGFR ≥40 mL/min/1.73 m2) and no evidence of secondary hypertension. Ambulatory blood pressure monitoring (ABPM) and renal function were assessed at baseline and three months after RDN. Patients were stratified into subgroups based on the number of ablation points (≤20 vs. >20). A two-tailed *p*-value <0.05 was considered statistically significant.

**Results:**

Data from eleven patients (mean age 64.4 ± 8.1, 45.5% were male) were analyzed. At three months, eGFR remained stable (mean decrease −4.6 mL/min/1.73 m^2^, CI 95% −15.8–6.6, *p* = 0.37). Mean ambulatory SBP and DBP decreased significantly following RDN (*Δ* −19.4mmHg, CI 95% −9.2 to −29.7, *p* = 0.002; *Δ* −8.6 mmHg, CI 95% −3.9 to −13.3, *p* = 0.002; for SBP and DBP respectively). Subgroup analysis revealed that patients with more ablation points experienced a greater reduction in SBP (*Δ* −17.7 mmHg, CI 95% 0.1–35.3, *p* = 0.04) and a numerically greater reduction in DBP (*Δ* −4.8 mmHg, CI 95% 4.5–12, *p* = 0.27) than those with fewer abation points.

**Conclusion:**

In this small retrospective cohort, reductions in SBP and DBP were observed following RDN using the SPYRAL™ multi-electrode system, with no excess of adverse renal events. These preliminary findings require confirmation in larger, adequately powered patient cohorts before firm conclusions can be established.

## Introduction

Arterial hypertension remains one of the most significant modifiable risk factors for cardiovascular morbidity and mortality worldwide (1). Despite the availability of numerous pharmacological therapies targeting different mechanisms, a substantial proportion of patients fail to achieve adequate blood pressure control, even when treated with combination therapy (2). Resistant hypertension, defined as uncontrolled blood pressure (BP) despite the use of at least three antihypertensive agents from different classes, including a diuretic, poses a considerable clinical challenge and is associated with an increased risk of cardiovascular and renal events (3, 4).

Renal sympathetic overactivity plays a central role in the pathophysiology of hypertension by modulating renin release, sodium retention, renal blood flow, and systemic vascular resistance (5). Catheter-based renal denervation (RDN) was developed as a device-based intervention to attenuate renal sympathetic nerve activity and thereby reduce blood pressure (6). Early unblinded trials demonstrated substantial long-term reductions in blood pressure (7); however, the neutral results of the SYMPLICITY HTN-3 trial raised concerns regarding patient selection, procedural technique, and the completeness of denervation (8). Subsequent studies using improved catheter designs and more comprehensive ablation strategies, including treatment of renal artery branch vessels, have reported significant reductions in ambulatory blood pressure (9, 10).

In addition to its blood pressure-lowering effects, renal safety remains a critical consideration, particularly in patients with multiple comorbidities. While meta-analyses of randomized trials have generally demonstrated preservation of renal function after RDN, real-world evidence remains limited (11). Moreover, the impact of the number and distribution of ablation points on procedural efficacy has not been fully elucidated (12, 13). Therefore, we aimed to investigate the effects of catheter-based RDN on ambulatory blood pressure and renal function in a real-world cohort and to explore whether the number of ablation points was associated with the magnitude of blood pressure reduction.

## Methods

### Study design and patient population

This retrospective, single-center observational study was conducted at the Department of Cardiology, Helios Kliniken Schwerin, Germany. The study adhered to the principles of the Declaration of Helsinki and aimed to evaluate real-world clinical outcomes in the first eleven consecutive patients treated with the Symplicity™ SPYRAL Renal Denervation System in Mecklenburg-Vorpommern, Germany between 2024 and 2025.

### Patient selection and inclusion criteria

Eligible participants were adults (≥18 years) with the following conditions: (i) resistant or uncontrolled arterial hypertension and/or (ii) documented intolerance to pharmacological therapy. Resistant hypertension was defined as persistent elevation in blood pressure (>140/90mmHg) despite a stable regimen of at least three antihypertensive drug classes, including a diuretic at the maximally tolerated dose. Exclusion criteria included: (i) secondary causes of hypertension (e.g., primary aldosteronism, pheochromocytoma); (ii) severely impaired renal function defined as estimated glomerular filtration rate (eGFR) < 40 mL/min/1.73 m^2^; and (iii) renal artery stenosis (>50%), fibromuscular dysplasia, or complex vascular anatomy as assessed by computed tomography (CT) or magnetic resonance (MR) angiography (14). In our analysis, the evaluation of resistant and secondary hypertension was guided by the 2024 ESC Guidelines for arterial hypertension (4).

### Renal denervation procedure

All procedures were performed using the Medtronic Symplicity™ SPYRAL Renal Denervation System under conscious sedation with intravenous propofol and remifentanil. Femoral arterial access was obtained under ultrasound guidance. Initial angiography was performed using a pigtail catheter in the abdominal aorta, followed by selective renal angiography with a 55 cm IMA catheter. A high-torque BMW guidewire (Abbott Vascular) was advanced into each renal artery, and radiofrequency ablations were applied sequentially from distal to proximal along the main renal artery. When anatomically feasible, additional ablations were delivered within renal artery side branches with a diameter >2.5 mm. Intravenous heparin was administered to maintain therapeutic anticoagulation. Hemostasis at the femoral access site was achieved using an Angio-Seal™ VIP vascular closure device (Terumo Europe).

### Procedural assessment of number of ablation points

For the exploratory procedural analysis, the number and anatomical distribution of documented radiofrequency ablation points were retrospectively extracted from the procedural records. Ablation points were categorized according to the treated renal arterial segment as follows: right and left main renal artery and right and left renal artery branch vessels. The total number of ablation points represented the sum of documented ablations delivered in these segments. The number and distribution of ablation points were determined according to the operator's assessment of the renal arterial anatomy and procedural feasibility.

### Data collection and follow-up

Demographic characteristics, cardiovascular risk factors, baseline antihypertensive therapy and therapy after RDN at the follow up, and procedural parameters were extracted from patients’ medical records. Medication adherence was assessed based on patient-reported medication use and review of the medication regimen at follow-up. Peri-procedural renal safety during the hospital stay was evaluated clinically using 24-hour urine output tracking post-procedure as the most appropriate parameter for early detection of possible kidney injury, followed by long-term eGFR (utilizing CKD-EPI equation) assessment during outpatient follow-up. Ambulatory 24-hour ambulatory blood pressure monitoring (ABPM) were assessed at baseline and three months after the RDN. BP was measured in the seated position after 5 min of rest, using a validated professional digital device (HBP-1100; Omron Healthcare, Kyoto, Japan) with an appropriate cuff size. ABPM was programmed to measure and record BP at 20-minute intervals during the day (06:00–22:00) and at 30-minute intervals during the night (22:00–06:00). Only recordings with a minimum of 80% valid readings were included in the final analysis. Procedural parameters, including procedure duration, radiation exposure, contrast volume, and total number of ablation points, were systematically recorded. Periprocedural access-site complications like hematoma, active bleeding, pseudoaneurysm; ii) vascular complications like renal artery dissection, stenosis or perforation; iii) hemodynamic complications like bradycardia or hypotension have been extracted from the records.

### Statistical analysis

Data management was conducted using Microsoft Excel (Microsoft Corp., Redmond, WA, USA), and all statistical analyses were performed using GraphPad Prism version 9.0 (GraphPad Software, La Jolla, CA, USA) and IBM SPSS Statistics for Windows, version 19.0 (IBM Corp., Armonk, NY, USA SPSS). Continuous variables are expressed as mean ± SD or median (IQR), as appropriate. Differences between groups of interest are reported as mean difference ± 95% CI or median (IQR), as appropriate. Normality of distribution for all continuous parameters (including ABPM and eGFR) was evaluated using Shapiro–Wilk and Kolmogorov–Smirnov tests combined with Q-Q plot inspection.

Paired baseline and 3-month outcome were compared using paired t-tests or Wilcoxon signed-rank tests, as appropriate. For exploratory analysis, patients were stratified into Group A (≤20 ablation points) and Group B (>20 ablation points) based on cohort data distribution rather than a prespecified clinical threshold. Between-group differences were explored using unpaired t-tests or Mann–Whitney U tests, or Fisher's exact tests, as appropriate. A two-tailed p-value <0.05 was considered statistically significant. For present analysis no formal sample size or power calculation could be applied due retrospective design with small sample size.

## Results

### Baseline characteristics

Eleven patients who underwent RDN—nine for true resistant hypertension and two due to medication intolerance. All received bilateral RDN, except for one patient whose procedure was stopped due to persistent restlessness (Supplement Table S1). Following the 3-month assessment, all antihypertensive medication types and dosages remained completely unchanged (Supplement Table S2). The mean age was 64.4 ± 8.1 years, with no significant difference of 5.7 years (CI 95% −5.1–16.6, *p* = 0.26) between group A (mean age 67.6 ± 5) and group B (mean age 61.8 ± 9.6) (Supplement Table S3).

Comorbidities were similarly distributed across groups. Obesity was present in 72.7% (*n* = 8) of patients (80.0% in group A vs. 66.7% in group B; *p* = 1.00). Coronary heart disease occurred in 27.2% (*n* = 3), all in group B (*p* = 0.18). Diabetes mellitus affected 36.3% (*n* = 4) of patients (20.0% in group A vs. 50.0% in group B, *p* = 0.54). Smoking was reported in 27.2% (*n* = 3), exclusively in group B (*p* = 0.18). No baseline demographic or clinical characteristics differed significantly between the groups (Table 1).

**Table 1:** Baseline demographic and clinical characteristics of the study population.

Characteristic	Total Cohort (*n* = 11)	Group A (≤ 20 Sites, *n* = 5)	Group B (> 20 Sites, *n* = 6)	*p*-Value
Gender, *n* (%)				0.567^a^
Male	5 (45.5)	3 (60.0)	2 (33.3)	
Female	6 (54.5)	2 (40.0)	4 (66.7)	
Age (years), mean ± SD	64.4 ± 8.1	67.6 ± 5	61.8 ± 9.6	0.26^b^
Comorbidities, *n* (%)				
Obesity	8 (72.7)	4 (80.0)	4 (66.7)	1.00^a^
Coronary heart disease	3 (27.3)	0 (0.0)	3 (50.0)	0.18^a^
Diabetes Mellitus	4 (36.4)	1 (20.0)	3 (50.0)	0.54^a^
Nicotine Abuse	3 (27.3)	0 (0.0)	3 (50.0)	0.18^a^

SD, standard deviation. Statistical significance was defined as *p* < 0.05.

a Fisher's exact test.

b Independent t-test.

### Blood pressure outcomes

After three months of follow-up, the significant reduction of ABPM following RDN was observed. Mean systolic blood pressure (SBP) decreased from 164.3 mmHg ± 17.7 at baseline to 144.8 mmHg ± 22.3 (*p* = 0.002) (Supplement Table S4). Similarly, mean diastolic blood pressure (DBP) decreased from 80.8 mmHg ± 14.9–72.2 mmHg ± 14.4 (*p* = 0.002) (Supplement Table 5). These findings indicate a notable reduction of −19.4 mmHg (95% CI −9.2 to −29.7 mmHg, *p* = 0.002) in SBP and −8.6 mmHg (CI 95% −3.9 to −13.3 mmHg, *p* = 0.002) in DBP during the study period (Figure 1) (Supplement Tables S4, S5).

**Figure 1:**
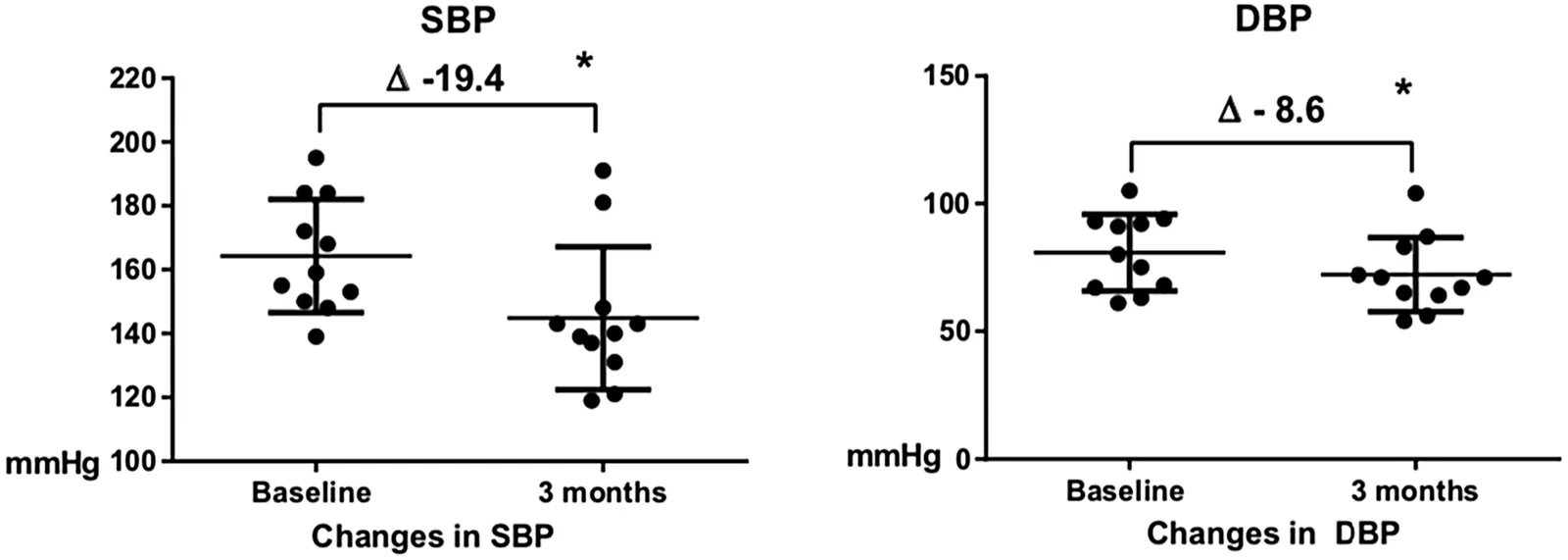
Mean changes in systolic (top left) and diastolic blood pressure (top right) three months following renal denervation; SBP, systolic blood pressure; DBP, diastolic blood pressure. *p < 0.05.

### Subgroup analysis based on number of ablation points

In group A, the mean SBP decreased from a baseline of 157.4 mmHg ± 21.8–147.6 mmHg ± 24.5 following RDN. In group B, the mean SBP decreased from 170.0 mmHg ± 12.7–142.5 mmHg ± 22.4. The mean differences for groups A and B were −9.8 mmHg (95% CI −15.2 to −4.3, *p* = 0.007) and −27.5 mmHg (95% CI −45.0 to −9.9, *p* = 0.01), respectively. Patients from group B experienced more significant SBP reduction in comparison with patients from group A (mean −17.7 mmHg, CI 95% −35.3 to −0.1, *p* = 0.04) (Figure 2). There was no significant difference of baseline SBP values between group A and B (mean difference 12.6 mmHg, CI 95% −11.1–36.3, *p* = 0.26). The number of ablations did not significantly correlate with absolute SBP reduction (Spearman r = 0.32, CI 95% −0.3–0.7, *p* = 0.32).

**Figure 2:**
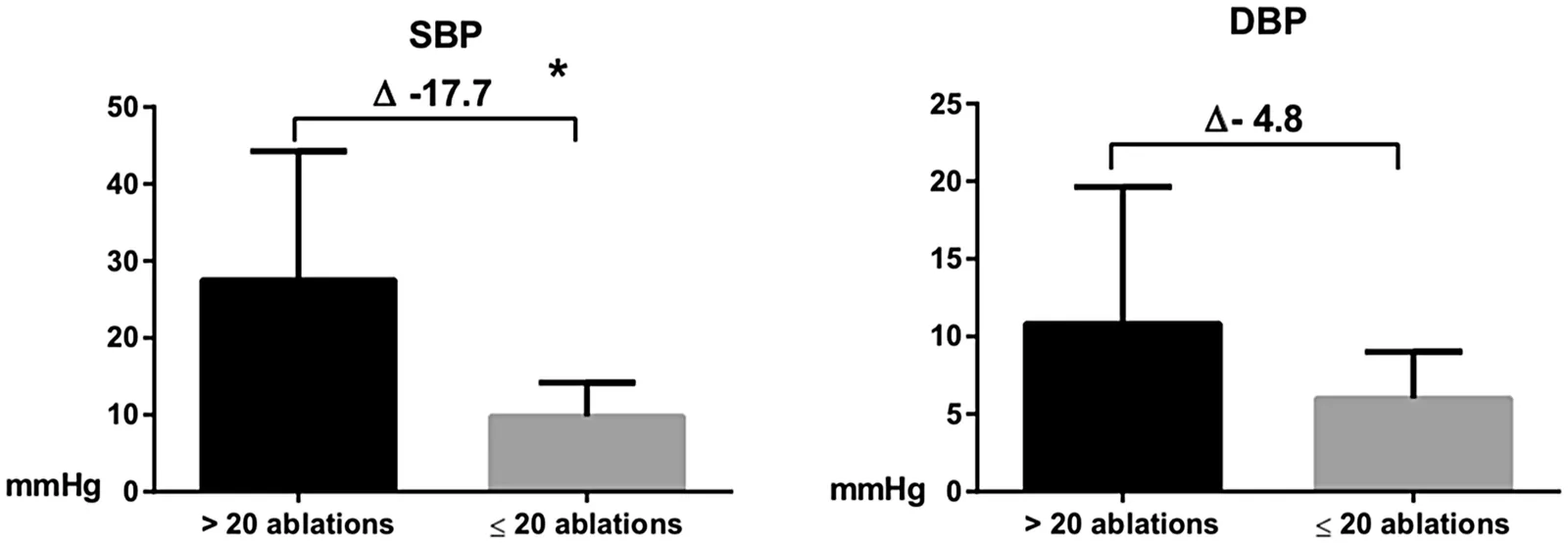
Differences in the reduction of systolic (top left) and diastolic (top right) blood pressure depending on the density of the applied ablation points; SBP, systolic blood pressure; DBP, diastolic blood pressure. *p < 0.05.

In group A, the mean DBP decreased from a baseline of 75.4 mmHg ± 15.8–69.4 mmHg ± 15.1 following RDN. In group B, the mean DBP decreased from 85.3 mmHg ± 13.8–74.5 mmHg ± 14.8. The mean differences for groups A and B were −6 mmHg (CI 95% −9.7 to −2.2, *p* = 0.01) and −10.8 mmHg (CI 95% −20 to −1.6, *p* = 0.02), respectively. There was no relevant difference concerning decrease of DBP between group A and B (mean −4.8 mmHg, CI 95% −14.2–4.5, *p* = 0.27) (Figure 2). There was no significant difference concerning baseline DBP values between group A and B (mean difference 9.9 mmHg, CI 95% −10.3–30.2 mmHg, *p* = 0.29).

Renal function remained stable in both groups after 3 months thereby showing slightly decrease of eGFR in group A (mean decrease of - 3.2 mL/min/1.73m2, CI 95% −25.1–18.6, *p* = 0.69) and in group B (mean decrease of −5.7 mL/min/1.73m2, CI 95% −24.1–12.6, *p* = 0.45). There was not significant difference concerning change of renal function between group A and B (- 2.4 mL/min/1.73m2, CI 95% −21.6–26.5, *p* = 0.82) (Supplement Table S3).

There was a significant difference regarding number of ablation points between group A (mean 16.6 ± 3.9) and group B (mean 24.7 ± 2.1). Patients in group B had in average 8 (CI 95% 3.9–12.2, *p* = 0.001) ablation points more compared with group A.

### Radiation dose, fluoroscopy/procedure time, and contrast agent’s volume

There were no significant differences in procedural metrics between the two groups. The applied radiation dose was numerically higher in group A compared with group B, though this did not reach statistical significance (group A median 1827 mGy, IQR 1204–6507 mGy vs. group B median 896 mGy, IQR 614–2184 mGy, *p* = 0.06). The volume of applied contrast dye did not differ significantly between group A and group B (median 200 mL, IQR 180–200 mL vs. median 50 mL, IQR 40–195 mL, *p* = 0.07). Similarly, no statistically significant difference was observed in procedure duration, although group A showed a numerically longer duration than group B (group A mean 19.2 min ± 7.3 vs. group B mean 13.2 min ± 4.6, *p* = 0.12) (Table 2).

**Table 2:** Procedural characteristics and comparison between group A and group B.

Procedural Parameter	Total Cohort (*n* = 11)	Group A (≤20 points, *n* = 5)	Group B (>20 points, *n* = 6)	*p*-Value
Number of ablation points (n)	21 ± 5.1	16.6 ± 3.9	24.7 ± 2.1	**<0.001** ^a^
Procedure duration (min)	16.5 ± 6.5	19.2 ± 7.3	13.2 ± 4.6	0.12^a^
Radiation dose (µGy · m^2^)	1007 IQR 787–2903	1827 IQR 1204–6507	896 IQR 614–2184	0.06^b^
Contrast medium (mL)	180 IQR 40 -200	200 IQR 180–200	50 IQR 40–195	0.07^b^

Bold value indicates statistical significance (*p* < 0.05).

a Unpaired T test.

b Man Whitney U test.

### Renal function and safety aspects

No patient met criteria for periprocedural acute kidney injury (urine-output less than 0.5 mL/kg/h over 6 h). Renal function remained stable after RDN, with mean value eGFR of 75.1 mL/min/1.73m2 ± 15.6 at baseline and 3 months following RDN eGFR of 70.5 mL/min/1.73m2 ± 20.3 (Supplement Table S3). There was no significant reduction of eGFR of - 4.6 mL/min/1.73m2 (CI 95% −15.8–6.6 mL/min/1.73m2, *p* = 0.38) following RDN in comparison with baseline values (Figure 3). None of the patients experienced vascular complications like access-site hematoma, pseudoaneurysm, renal artery stenosis or perforation, and/or hemodynamic deterioration in term of severe bradycardia or hypotension.

**Figure 3:**
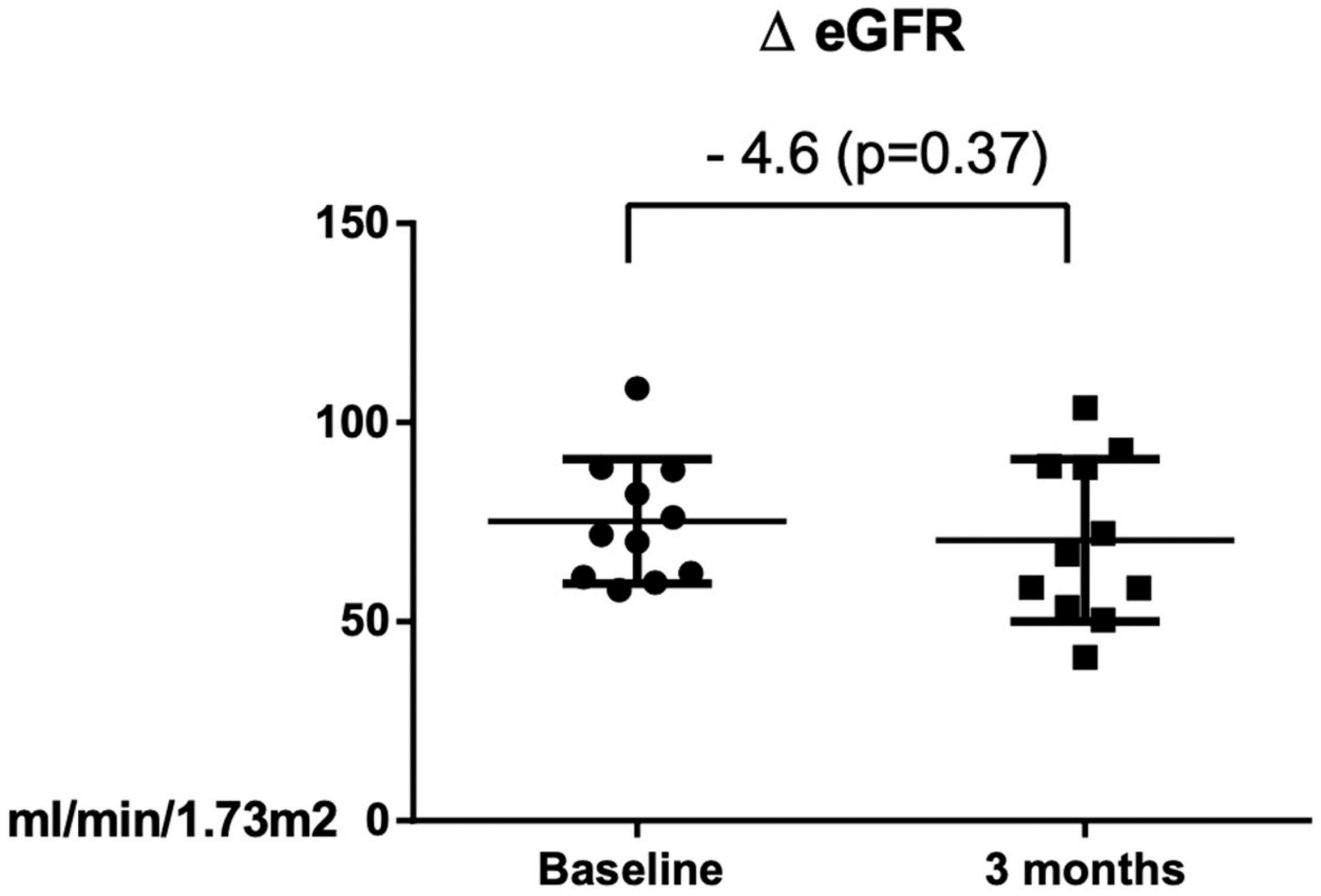
Change of renal function based on estimated glomerular filtration rate (eGFR) following renal denervation.

## Discussion

In this small, single-center, real-world cohort, reductions in both systolic and diastolic ABPM were observed three months after catheter-based RDN with the SPYRAL™ multi-electrode system without any renal safety concerns. Furthermore, preliminary data from this small sample suggest that a higher number of ablation points may yield a greater pressure drop, a signal that should be further scrutinized in adequately powered prospective studies.

Our findings are consistent with prior randomized trials, including the SPYRAL HTN-OFF MED and ON MED programs, which established RDN as a safe and effective adjunct therapy for hypertension management (13, 15).

### Hemodynamic efficacy and clinical relevance

In this uncontrolled cohort, we observed reduction of SBP by 19.4 mmHg (Figure 1) and DBP by 8.6 mmHg (Figure 2) at three months after RDN procedure. Due to limited statistical power, this observation is rather descriptive and cannot be definitely attributed do RDN itself. Though, a patient-level meta-analysis of 48 trials involving more than 340,000 patients showed that a 5-mmHg reduction in SBP is associated with a 10% decrease in major cardiovascular events (16), thus further highlights the possible potential of RDN to reduce global cardiovascular risk, especially in patients with treatment-resistant hypertension (3, 4).

### Exploratory subgroup analysis of outcomes by ablation point volume

Our exploratory subgroup analysis was conducted to descriptively compare patients receiving fewer than 20 ablation points against those receiving more than 20 points. Patients with a higher number of radiofrequency ablations achieved greater reduction in SBP (−27 mmHg) and numerically greater DBP reduction (−10 mmHg) compared to those with fewer ablation points (−10 mmHg for SBP and −6 mmHg for DBP). This finding is consistent with previous evidence suggesting that more comprehensive and circumferential ablation coverage may be the key determinant of procedural efficacy (17, 18). Furthermore, a higher number of ablation points was associated with lower burden concerning radiation exposure (*p* = 0.06) and reduced amount of contrast dye (*p* = 0.07), although these relationships reached only borderline statistical significance. Although the small sample size does not allow for a definitive conclusion, it appears from these data that more extensive ablation is not associated with greater complications. Of note, the numerically higher baseline SBP in Group B compared to Group A, combined with the lack of a significant correlation between the number of ablation points and absolute SBP reduction, suggests that our findings may be confounded. Furthermore, the role of regression to the mean cannot be ruled out.

Evidence from *post-hoc* evaluations of large-scale RDN trials further supports the hypothesis that the total number of ablations significantly influences hemodynamic outcomes. In these analyses, a higher frequency of ablation points was consistently associated with more robust blood pressure lowering, whereas insufficient number of ablations was linked to suboptimal or non-responder status. Notably, data have shown that patients receiving fewer than nine ablations often exhibit little to no clinical benefit, while those treated with more than fourteen points demonstrate significantly enhanced antihypertensive responses (16, 19).

Modern RDN strategies, particularly those utilizing the multi-electrode SPYRAL system, emphasize intensive ablation techniques that include the treatment of distal renal artery branches. This approach is anatomically grounded, as sympathetic nerves are known to be situated in closer proximity to the arterial lumen in more distal segments (13, 19). Furthermore, procedural techniques ensuring circumferential ablation—targeting all four quadrants of the vessel—have been associated with superior blood pressure responses. This underscores the necessity of comprehensive, 360-degree nerve disruption rather than limited, focal ablation to achieve a consistent sympathetic block (15, 19).

Paradoxically, the group with fewer ablations required higher radiation doses and contrast volumes. These findings likely reflect greater procedural complexity, such as challenging catheter engagement or positioning based on individual renal artery anatomy. Furthermore, patient obesity may have increased technical difficulty and fluoroscopy requirements. Taken together, these anatomical and procedural challenges may explain variability between groups, suggesting that patients with fewer ablations probably presented with more challenging anatomy.

However, it is important to acknowledge that the relationship between the number of ablation points and treatment response is not universally linear. In some observational cohorts, responder status was not significantly correlated with the absolute number of ablations performed (11, 20). This suggests that while ablation density is a critical procedural factor, clinical outcomes are likely multifactorial, influenced by baseline sympathetic activity, individual vascular anatomy, patient-specific characteristics, and the operator's experience in achieving effective wall contact (21). Therefore, it is of paramount importance to perform a comprehensive patient's selection potentially eligible for RDN in order to identify responder and differentiate from non-responders as this may have important clinical implications (22). There are some data suggesting that the presence of arterial stiffness predicts an inadequate blood pressure response after RDN (23). Heart rate above 73.5 beats per minute was associated with better BP response following RDN in a SPYRAL HTN-OFF MED trial (24). Renal nerve stimulation induced BP increase may be a predictor of adequate BP reduction following RDN (25).

### Renal function

Renal safety remains a key consideration in interventional treatment of hypertension. In this cohort, mean eGFR remained stable, and no patient experienced acute kidney injury or deterioration in renal function. These findings are consistent with long-term registry data, including the Global SYMPLICITY Registry, which demonstrate preserved renal function in patients with baseline normal kidney function following RDN (11).

### Strengths and limitations

This study provides valuable real-world evidence on the implementation of multi-electrode RDN. Strengths include its practical clinical setting, comprehensive assessment of both hemodynamic and renal safety outcomes, and evaluation of procedural factors such as ablation frequency. However, several key limitations must be acknowledged. The primary limitations of this study, namely its small sample size (*n* = 11), retrospective single-center design, short follow-up duration, and heterogeneous indications (nine resistant vs. two drug-intolerant patients), collectively restrict its statistical power and the generalizability of the findings. Due to the absence of a control group and objective pharmacological testing of therapy adherence, the drop in BP could also be partially explained by other factors like better patient monitoring, improving adherence to therapy, and not exclusively through the RDN procedure itself. Moreover, these findings are specific to RDN performed using the SPYRAL™ system only. Therefore, this analysis and its findings are rather exploratory and hypothesis-generating that should be interpreted with caution.

## Conclusion

In this small retrospective cohort, reductions in SBP and DBP were observed following RDN using the SPYRAL™ multi-electrode system, with no excess of adverse renal events. Ultimately, establishing firm conclusions and a clearer understanding of the factors driving successful blood pressure reduction will require validation in larger, adequately powered patient cohorts.

## Data Availability

The raw data supporting the conclusions of this article will be made available by the authors, without undue reservation.

## References

[1] BD 2019 Risk Factors Collaborators. Global burden of 87 risk factors in 204 countries and territories, 1990–2019. Lancet. (2020) 396:1223–49. 10.1016/S0140-6736(20)30752-2 33069327 PMC7566194

[2] Zhou B Carrillo-Larco RM Danaei G Riley LM Paciorek CJ Stevens GA. Worldwide trends in hypertension prevalence and progress in treatment and control from 1990 to 2019. Lancet. (2021) 398:957–80. 10.1016/S0140-6736(21)01330-1 34450083 PMC8446938

[3] Carey RM Calhoun DA Bakris GL Brook RD Daugherty SL Dennison-Himmelfarb CR. Resistant hypertension: detection, evaluation, and management: a scientific statement from the American Heart Association. Hypertension. (2018) 72:117–30. 10.1161/HYP.0000000000000084 PMC6530990 30354828

[4] Williams B Mancia G Spiering W Agabiti Rosei E Azizi M Burnier M. 2018 ESC/ESH guidelines for the management of arterial hypertension. Eur. Heart J. (2018) 39:3021–104. 10.1093/eurheartj/ehy339 30165516

[5] DiBona GF. Physiology in perspective: the wisdom of the body. Neural control of the kidney. Am J Physiol. (2005) 289:R633–41. 10.1152/ajpregu.00258.2005 16105818

[6] Esler M Krum H Sobotka PA Schlaich M Schmieder RE Böhm M. Renal sympathetic denervation in patients with treatment-resistant hypertension. Lancet. (2010) 376:1903–9. 10.1016/S0140-6736(10)62039-9 21093036

[7] Krum H Schlaich M Whitbourn R Sobotka PA Bartus K Straznicky N. Percutaneous renal denervation in patients with treatment-resistant hypertension: final 3-year report of the symplicity HTN-1 study. Lancet. (2014) 383:622–9. 10.1016/S0140-6736(13)62192-3 24210779

[8] Bhatt DL Kandzari DE O'Neill WW D'Agostino R Flack JM Katzen BT. A controlled trial of renal denervation for resistant hypertension. N Engl J Med. (2014) 370:1393–401. 10.1056/NEJMoa1402670 24678939

[9] Townsend RR Mahfoud F Kandzari DE Kario K Pocock S Weber MA. Catheter-based renal denervation in patients with uncontrolled hypertension (SPYRAL HTN-OFF MED): a randomised, sham-controlled, proof-of-concept trial. Circulation. (2017) 135:110–20. 10.1016/S0140-6736(17)32281-X 28859944

[10] Böhm M Kario K Kandzari DE Mahfoud F Weber MA Schmieder RE. Efficacy of catheter-based renal denervation in the absence of antihypertensive medications: 6-month results from SPYRAL HTN-OFF MED. Lancet. (2020) 395:1444–51. 10.1016/S0140-6736(20)30554-7 32234534

[11] Vukadinović D Lauder L Kandzari DE Bhatt DL Kirtane AJ Edelman ER. Effects of catheter-based renal denervation in hypertension: a systematic review and meta-analysis. Circulation. (2024) 150:1599–611. 10.1161/CIRCULATIONAHA.124.069709 PMC11560572 39355923

[12] Azizi M Schmieder RE Mahfoud F Weber MA Daemen J Davies J. Endovascular ultrasound renal denervation to treat hypertension: primary results of the RADIANCE-HTN SOLO trial. Lancet. (2018) 391:2335–45. 10.1016/S0140-6736(18)31082-1 29803590

[13] Weber MA Osborn JW. Improved understanding of renal nerve anatomy: an opportunity to enhance denervation treatment of hypertension. JACC: Cardiovasc. Interv. (2021) 14(3):316–18. 10.1016/j.jcin.2020.11.003 33541542

[14] Mancia G Kreutz R Brunström M Burnier M Grassi G Januszewicz A. 2023 ESH guidelines for the management of arterial hypertension. J Hypertens. (2023) 41:1874–2071. 10.1097/HJH.0000000000003480 37345492

[15] Kandzari DE Böhm M Mahfoud F Townsend RR Weber MA Pocock S. Effect of renal denervation on blood pressure in the SPYRAL HTN-OFF MED trial. Circulation. (2018) 391:2346–55. 10.1016/S0140-6736(18)30951-6

[16] Rahimi K Bidel Z Nazarzadeh M Copland E Canoy D Ramakrishnan R. Flood pressure lowering treatment trialists’ collaboration. Pharmacological blood pressure lowering for primary and secondary prevention of cardiovascular disease across 48 trials. Lancet. (2021) 397:1625–36. 10.1016/S0140-6736(21)00590-0 33933205 PMC8102467

[17] Tzafriri AR Mahfoud F Keating JH Markham PM Spognardi A Wong G. Innervation patterns may limit response to renal denervation. J Am Coll Cardiol. (2014) 64:1079–87. 10.1016/j.jacc.2014.07.937 25212640 PMC4377282

[18] Roubsanthisuk W Kunanon S Chattranukulchai P Panchavinnin P Wongpraparut N Chaipromprasit J. 2022 Renal denervation therapy for the treatment of hypertension: thai hypertension society statement. Hypertens Res. (2023) 46:898–912. 10.1038/s41440-022-01133-6 36759658 PMC10073020

[19] Mahfoud F Serruys PW. Renal denervation reloaded: where to go from here? EuroIntervention. (2015) 10:1133–6. 10.4244/EIJV10I10A187 25701510

[20] Rohla M Nahler A Lambert T Reiter C Gammer V Grund M. Predictors of response to renal denervation for resistant arterial hypertension. J Hypertens. (2016) 34:123–9. 10.1097/HJH.0000000000000764 26556567

[21] Lewington S Clarke R Qizilbash N Peto R Collins R. Age-specific relevance of usual blood pressure to vascular mortality: a meta-analysis of individual data for one million adults in 61 prospective studies. Lancet. (2002) 360:1903–13. 10.1016/S0140-6736(02)11911-8 12493255

[22] Fengler K Reimann P Rommel KP Kresoja KP Blazek S Unterhuber M. Comparison of long-term outcomes for responders versus non-responders following renal denervation in resistant hypertension. J Am Heart Assoc. (2021) 10(21):e022429. 10.1161/JAHA.121.022429 34713718 PMC8751833

[23] Fengler K Rommel KP Kriese W Kresoja KP Blazek S Obradovic D. Assessment of arterial stiffness to predict blood pressure response to renal sympathetic denervation. Euro Intervention. (2022) 18(8):e686–94. 10.4244/EIJ-D-21-01036 35244604 PMC10241279

[24] Böhm M Mahfoud F Townsend RR Kandzari DE Pocock S Ukena C. Ambulatory heart rate reduction after catheter-based renal denervation in hypertensive patients not receiving anti-hypertensive medications: data from SPYRAL HTN-OFF MED, a randomized, sham-controlled, proof-of-concept trial. Eur Heart J. (2019) 40(9):743–51. 10.1093/eurheartj/ehy871 Erratum in: *Eur Heart J*. (2019) 40(27):2205. doi: 10.1093/eurheartj/ehz184. 30608521

[25] De Jong MR Adiyaman A Gal P Smit JJ Delnoy PP Heeg JE. Renal nerve stimulation-induced blood pressure changes predict ambulatory blood pressure response after renal denervation. Hypertension. (2016) 68(3):707–14. 10.1161/HYPERTENSIONAHA.116.07492 27432864

