# Supplementary material for: Early blood pressure outcomes after SPYRAL™ renal denervation: an exploratory small cohort analysis
Source: Front Cardiovasc Med. 2026 Oct 1;13:1934448. doi: 10.3389/fcvm.2026.1934448 (PMC13670513; doi:10.3389/fcvm.2026.1934448)
Supplement: Supplementary file 1 [file Supplementaryfile1.docx]

**Supplement**

**Table 1. Number of ablation points in the main and side branch vessels**

| **Patient** | **Left main artery (n)** | **Left branch vessel (n)** | **Right main**  **artery (n)** | **Right branch vessel (n)** |
| --- | --- | --- | --- | --- |
| **1.** | **1** | **5** | **5** | **0** |
| **2.** | **5** | **0** | **9** | **0** |
| **3.** | **11** | **5** | **5** | **5** |
| **4.** | **2** | **10** | **4** | **8** |
| **5.** | **12** | **0** | **10** | **0** |
| **6.** | **11** | **2** | **12** | **0** |
| **7.** | **8** | **0** | **12** | **0** |
| **8.** | **12** | **7** | **discontinuation** | **discontinuation** |
| **9.** | **7** | **3** | **0** | **9** |
| **10.** | **7** | **4** | **9** | **7** |
| **11.** | **6** | **2** | **8** | **7** |

**Table 2. Antihypertensive medication at baseline and following RDN**

| **Patient** | **Number of antihypertensive medications** |
| --- | --- |
| **1.** | Four-drug therapy (including diuretic + MRA) |
| **2.** | Three-drug therapy (including diuretic) |
| **3.** | Four-drug therapy (including diuretic + MRA) |
| **4.** | Three-drug therapy (including MRA) |
| **5.** | Four-drug therapy (including diuretic + MRA) |
| **6.** | Four-drug therapy (including diuretic + MRA) |
| **7.** | Four-drug therapy (including diuretic + MRA) |
| **8.** | Four-drug therapy (including diuretic + MRA) |
| **9.** | Two-drug therapy (including diuretic + MRA) |
| **10.** | Two-drug therapy (including diuretic + MRA) |
| **11.** | Four-drug therapy (including diuretic + MRA) |

**Table 3. Age and absolute values of eGFR at baseline and after 3 months**

| **Patient** | **Age** | **eGFR at baseline** | **eGFR at 3 months** | **Δ eGFR**  **after RDN** | **Ablation points (n)** |
| --- | --- | --- | --- | --- | --- |
| **1.** | **60** | **82** | **50.4** | **- 31.6** | **11** |
| **2.** | **67** | **70.1** | **66.9** | **- 3.2** | **14** |
| **3.** | **43** | **108.5** | **103.7** | **- 4.8** | **26** |
| **4.** | **62** | **61.2** | **53.6** | **- 7.6** | **24** |
| **5.** | **70** | **59.8** | **41** | **- 18.8** | **22** |
| **6.** | **67** | **88.7** | **58.4** | **- 30.3** | **25** |
| **7.** | **74** | **88** | **89** | **1** | **20** |
| **8.** | **69** | **57.9** | **58.5** | **0.6** | **19** |
| **9.** | **68** | **76.2** | **93** | **16.8** | **19** |
| **10.** | **63** | **62.2** | **72.2** | **10** | **28** |
| **11.** | **66** | **71.8** | **88.7** | **16.9** | **23** |
| **Mean (95% CI)** | **64.4**  **(59 to 69.9)** | **75.1**  **(64.6 to 85.6)** | **70.5**  **(56.8 to 84.2)** | **- 4.6**  **(-15.8 to 6.6)** |  |

**eGFR – estimated glomerular filtration rate**

**Table 4. Systolic blood pressure values at baseline and following RDN**

| **Patient** | **SBP at baseline**  **(mmHg)** | **SBP following RDN (mmHg)** | **Δ**  **(mmHg)** | **Ablation**  **points (n)** |
| --- | --- | --- | --- | --- |
| **1.** | **148** | **137** | **- 11** | **11** |
| **2.** | **155** | **139** | **- 16** | **14** |
| **3.** | **184** | **181** | **- 3** | **26** |
| **4.** | **159** | **143** | **- 16** | **24** |
| **5.** | **168** | **143** | **- 15** | **22** |
| **6.** | **172** | **121** | **- 51** | **25** |
| **7.** | **150** | **140** | **- 10** | **20** |
| **8.** | **195** | **191** | **- 4** | **19** |
| **9.** | **139** | **131** | **- 8** | **19** |
| **10.** | **184** | **148** | **- 26** | **28** |
| **11.** | **153** | **119** | **- 34** | **23** |
| **Mean (95% CI)** | **164.3**  **(152.4 to 176.2)** | **144.8**  **(129.8 to 159.8)** | **- 19.4 ***  **(-9.2 to -29.7)** |  |

**RDN –renal denervation; SBP – systolic blood pressure; CI – confidence interval;**

*** p <0.05**

**Table 5. Diastolic blood pressure values at baseline and following RDN**

| **Patient** | **DBP at baseline**  **(mmHg)** | **DBP following RDN (mmHg)** | **Δ**  **(mmHg)** | **Ablation points (n)** |
| --- | --- | --- | --- | --- |
| **1.** | **61** | **54** | **- 7** | **11** |
| **2.** | **92** | **83** | **- 9** | **14** |
| **3.** | **105** | **104** | **- 1** | **26** |
| **4.** | **67** | **65** | **- 2** | **24** |
| **5.** | **80** | **71** | **- 9** | **22** |
| **6.** | **94** | **72** | **- 12** | **25** |
| **7.** | **63** | **56** | **- 7** | **20** |
| **8.** | **93** | **87** | **- 6** | **19** |
| **9.** | **68** | **67** | **- 1** | **19** |
| **10.** | **91** | **71** | **- 20** | **28** |
| **11.** | **75** | **64** | **- 11** | **23** |
| **Mean (95% CI)** | **80.8**  **(70.8 to 90.9)** | **72.2**  **(62.5 to 81.9)** | **- 8.6 ***  **(-3.9 to 13.3)** |  |

**RDN –renal denervation; DBP – diastolic blood pressure; CI – confidence interval;**

*** p <0.05**

**Figure 1. Figure Scatterplot Ablation number against absolute SBP drop / Spearman correlation**
